# Leptin: role over central nervous system in epilepsy

**DOI:** 10.1186/s12868-018-0453-9

**Published:** 2018-09-05

**Authors:** Laura Mora-Muñoz, Alejandro Guerrero-Naranjo, Elisa Angélica Rodríguez-Jimenez, Claudio Alberto Mastronardi, Alberto Velez-van-Meerbeke

**Affiliations:** Escuela de Medicina y Ciencias de la Salud, Universidad del Rosario, Cra 24 No 63C-69, Bogotá, Colombia

**Keywords:** Leptin, Adipose tissue, Nervous system, Hypothalamus, Epilepsy, Anticonvulsants, Convulsants

## Abstract

Adipose tissue is a dynamic organ with different effects on the body. Many of these effects are mediated by leptin, a hormone strongly involved in regulation of feeding and energy metabolism. It has an important role as a mediator of neuronal excitatory activity and higher brain functions. The aim of this study was to review the association between leptin and cerebral neuronal function, in particular its anticonvulsant or convulsant effects and the possible therapeutic role for treating epilepsy. For this purpose, the databases Pubmed, Science Direct, Elsevier, ResearchGate and Scielo were searched to identify experimental studies, reviews and systematic review articles, published in English, Spanish or Portuguese. Experimental studies and the presence of leptin receptors in nervous system sites other than the hypothalamus suggest an influence on higher brain functions. Indeed several animal studies have demonstrated a role of these channels in epileptiform activity as both anticonvulsive and convulsive effects have been found. The reason for these discrepancies is unclear but provides clear evidence of a potential role of leptin and leptin therapy in epileptiform activity. The association between leptin and brain function demonstrates the importance of peripheral metabolic hormones on central nervous system and opens a new way for the development of novel therapeutic interventions in diseases like epilepsy. Nevertheless further investigations are important to clarify the dynamics and diverse actions of leptin on excitatory regulation in the brain.

**Electronic supplementary material:**

The online version of this article (10.1186/s12868-018-0453-9) contains supplementary material, which is available to authorized users.

## Background

The central nervous system (CNS), specifically the hypothalamus, acts as a sensor of storage and energy expenditure. Adipocytes, the most important functional unit of adipose tissue, secrete adipokines, such as leptin, that regulate energy balance [1]. Although the role of leptin at the hypothalamic level to regulate food intake and energy balance is well established, its participation on higher brain functions remains to be elucidated. For instance, there is evidence showing that leptin can act both as anticonvulsant and convulsant trigger [1].

Epilepsy is a chronic neurological disorder experienced by approximately fifty million people around the world. Its prevalence in the general population is 4–10 per 1000 people, while in low and middle-income countries is higher (7–14 per 1000 people) [2]. Whereas active epilepsy (defined as a history of more than one unprovoked seizure and either recent seizures in the previous 5 years or current use of antiepileptic medication), is 12.4 per 1000 people ranging from 5.1/1000 to 57/1000 [3–5]. The incidence of epilepsy range between 30 and 50 per 100,000. In 2012, 20.6 billion disability adjusted life years (DALYs) were due to epilepsy [2].

The objective of this article was to analyze the possible effects of leptin on the pathophysiology of epilepsy, comparing the findings of animal models, which demonstrated both convulsive and non-convulsive actions. To contextualize the review, we described the molecular characteristics of leptin, its various isoforms, the receptors in the central nervous system, the signaling pathway in nervous cells, the role and functions of leptin within the CNS affecting higher brain functions, and/or showed the specific actions related to the mechanism of epilepsy.

A review of literature was carried out. The following databases were consulted: Pubmed, Science Direct, Elsevier ResearchGate and Scielo. Eligibility criteria were: experimental studies, reviews and systematic review articles; that were selected if the publication date between January 1997 and January 2018; restricted to non-human studies and articles published in English, Spanish and Portuguese. Research was made through the Medical Subject Headings (MESH) terms: MeSH keywords in Pubmed: (((“Epilepsy”[Mesh]) AND “Leptin”[Mesh]) OR “Adipose Tissue”[Mesh]) OR “Hypothalamus”[Mesh]. The same search terms were adapted for Science Direct, Elsevier ResearchGate and Scielo electronic databases. The corresponding Health Sciences Descriptors in Spanish (DeCS) terms were also used. Exclusion criteria were studies outside the range of proposed dates and studies made in humans.

**Fig. 1:**
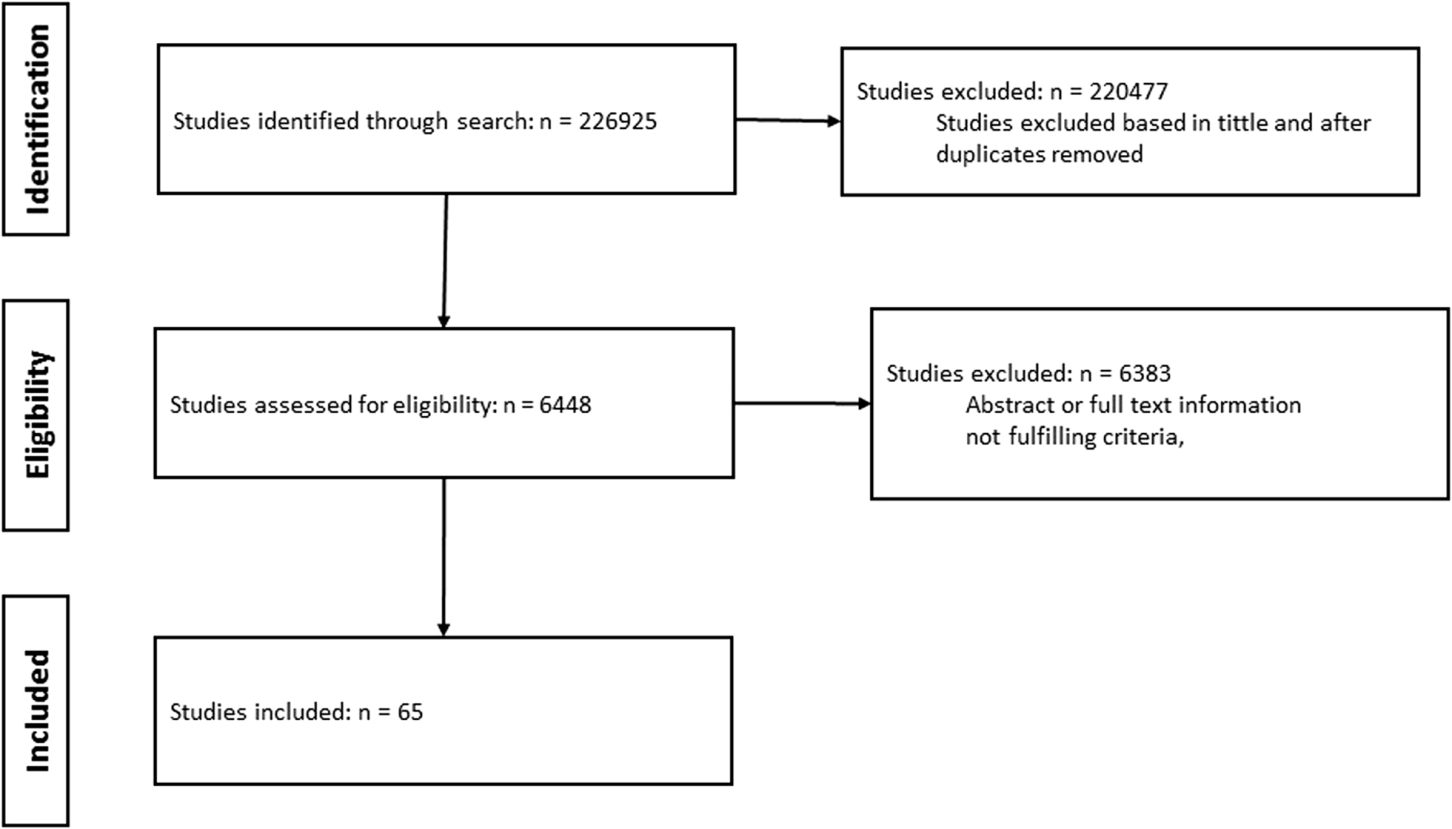
Review flow diagram

Two authors (LMM, AGN) independently evaluated the eligibility of all studies by checking the title and the abstract to determine whether they met all of the inclusion criteria. Disagreements were resolved by discussion or consultation with other authors (AVM). When they were ambiguous, the complete articles were analyzed to determine their pertinence. A total of 226,925 articles were identified through databases searching using the MESH an DECS terms (Fig. 1). Consecutively, the articles were selected based on title, abstract, and, in text information. Based on these criteria, a total of 65 articles were chosen.

As a meta-analysis was not performed due to the characteristics and heterogeneity of the articles, a narrative report was done extracting the most important information of each article.

## Leptin: a pleiotropic hormone

Leptin, a 16-kDa protein encoded by the Ob gene, regulates body weight, reduces appetite, and controls food intake. Insulin, glucocorticoids, leptin and other hormones regulate expression of this protein. It appears that several metabolic actions are controlled through FOSL2, a key transcription factor that regulates leptin expression in the adipocyte [6–8].

**Fig. 2:**
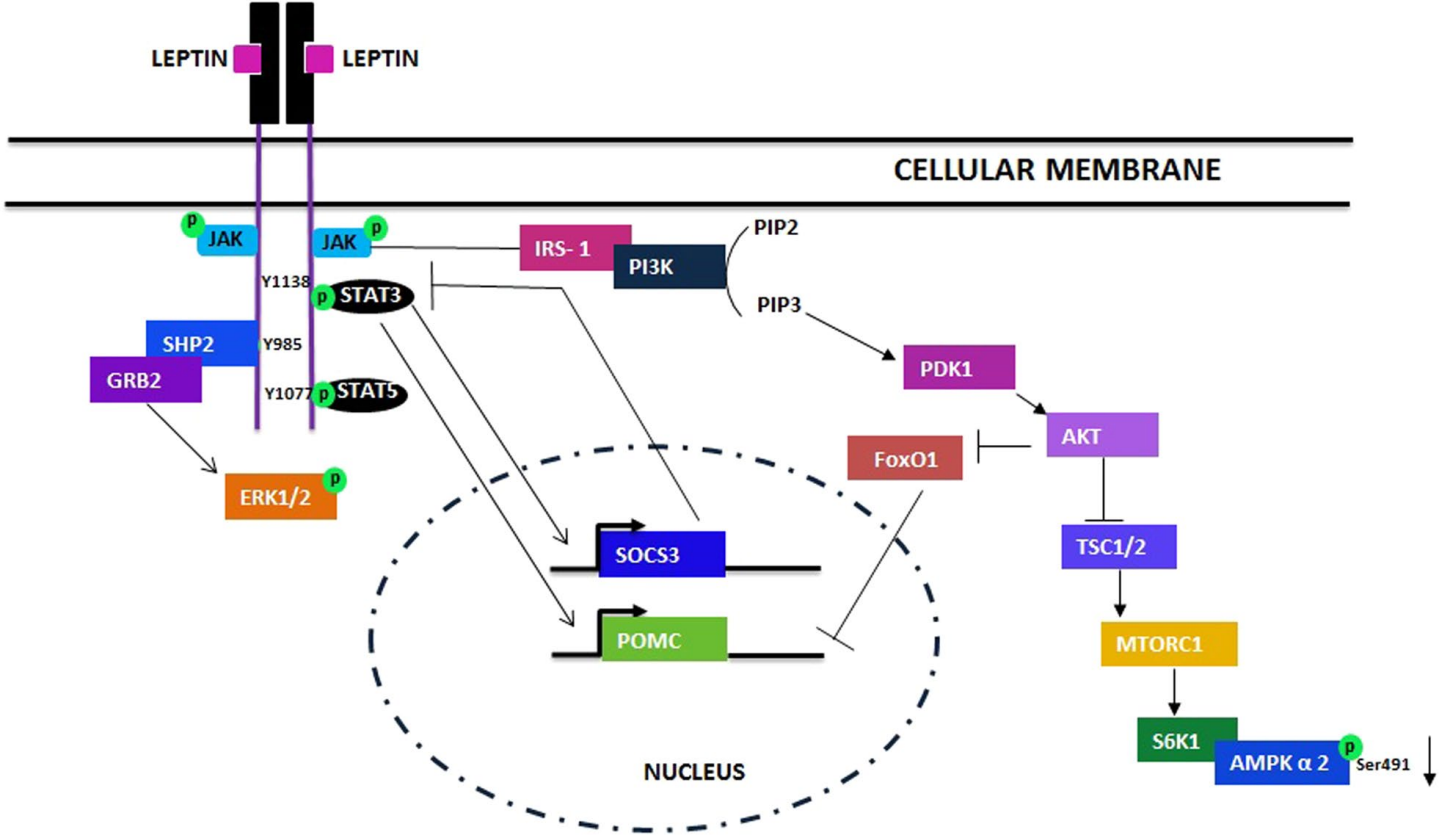
Leptin intracellular signaling pathway

There are six isoforms of the leptin receptor (Ob-Ra, Ob-Rb, Ob-Rc, Ob-Rd, Ob-Re and OB-Rf) that result from RNA alternative splicing. According to their domains, are classified in short, long and secreted [9]. Ob- Rb stands out as the only receptor type showing a full-length intracellular domain containing 304 residues, which gives it the ability to activate intracellular signaling cascades. Once leptin binds to Ob-Rb, it elicits the auto-phosphorylation of Janus kinase -2 (JAK- 2), a non-receptor tyrosine kinase that is associated with the Ob-Rb receptor. Thereafter, JAK- 2 phosphorylates tyrosine (Tyr) residues of the intracellular domain of Ob-Rb, and depending on the Tyr residue that is phosphorylated it can trigger different types of intracellular responses [1, 9] (Fig. 2).

In the case that Tyr 985 is phosphorylated, it will lead to the recruitment of Src homology 2 domain (SH2), triggering a signaling cascade mediated by extracellular signal-regulated kinase (ERK) [9]. The SH2-containing tyrosine-specific protein phosphatase (SPH2), and “SH2 domain-containing adaptor, the growth factor receptor-binding protein” (GRB2), enable ERKs phosphorylation [10]. Its activation has been related to energy homeostasis, as long as its blockage incurs in a disruption in the induction of sympathetic activation of BAT and a deregulation of its anorexic, and weight controlling actions [11].

On the other hand, if Tyr 1077 residue is phosphorylated, it leads to signal transducer and activator of transcription 5 (STAT -5) activation, whereas if Tyr 1138 residue is phosphorylated, it will lead to STAT 3 activation. STAT phosphorylation leads to the dimerization and its subsequent translocation to the nucleus, coordinating the transcription of genes involved in energy balance such as proopiomelanocortin (POMC) [1, 9]. POMC is a protein that can be cleaved to render the anorexigenic α-melanocyte stimulating hormone (α -MSH), which constitutes the major target of this pathway [9]. It is noteworthy to remark that STAT 3 also plays a significant role in controlling the transcription of genes that are implicated in leptin signaling, such as suppressor of cytokine signaling 3 (SOCS3), a key negative regulator of leptin effects, by inhibiting leptin- STAT3 signaling [12].

Activation of phosphatidylinositol-4, 5-bisphosphate 3-kinase (PI3K) by the Insulin Receptor Substrate complex, is also induced. PI3 K mediates the signaling of leptin in hypothalamic neurons, regulating neuro electrical effects on POMC neurons. This enzyme phosphorylates PIP2 (phosphatidylinositol-4,5-bisphosphate) and forms PIP3 (phosphatidylinositol-3,4,5-triphosphate), which links the activity of PI3 K with important downstream pathways, like Akt-FoxO1 and Akt-mTOR [9]. Interestingly, as demonstrated, leptin can inhibit different forms of epileptiform-like activity via PI3 K pathway [13].

The activity of Akt within the Akt- mammalian target of rapamycin1 (mTORC1) pathway allows the activation of mTORC1 mainly by various mechanisms that have been subject to extensive study. mTOR activation is related with leptin action in the hypothalamus [14]. Leptin increases hypothalamic mTOR activity, and this the serine-threonine kinase, through its downstream target Ribosomal protein S6 kinase beta (S6K1), phosphorylates α2-AMPK. In consequence the activity of leptin decreases food intake and body weight. Additionally, mTOR mediates the anorexigenic, sympathetic and cardiovascular effects of leptin [9].

## Leptin and central nervous system

As it was mentioned above, leptin plays key roles within the hypothalamus to control food intake and energy expenditure. However, there is also evidence suggesting that leptin could be involved in other neurological processes in extra-hypothalamic sites. Interestingly, within the hippocampus leptin was associated with both anticonvulsant and convulsant effects, and it was also shown to act as neurotrophic factor that promotes neurogenesis [7, 15–17].

Leptin has been strongly associated with the regulation of neuronal excitability in different regions of the CNS [18, 19]. It modulates cell excitation by activating large conductance *Ca*^+^-*activated potassium channels (*BK channels) within the hippocampus and by stimulating *ATP*-*sensitive potassium channels (*KATP channels) during its regulatory metabolic functions within the hypothalamus and insulin-secreting cells [20–22]. The latter, highlights the anorexigenic leptin functions that are well described in the literature. However, mounting evidence also suggest other central actions of leptin in multiple sclerosis, amyotrophic lateral sclerosis, Alzheimer, that remain to be better understood [23–25].

There is evidence showing association between leptin and high brain functions, such as cognition and memory; for example, higher leptin levels are associated with a lower risk of incident dementia and Alzheimer’s disease (AD) [26]; leptin improves memory and serves as disease modifying therapeutic in transgenic mouse models of AD [27]; leptin receptor-deficient rodents have impairments in spatial memory and alterations in LTP and LTD in hippocampal CA1 neurons[28]; physiological increases in leptin levels facilitate the CaMK II activity in the hippocampus, which induces LTP, contributing to memory and learning[29]; and leptin facilitate spatial learning and hippocampal LTP in mice [30]. In vivo studies in rodents reported that leptin can increase the proportion of dendrites enabling morphological changes that can promote the formation of new synaptic connections within the hippocampus [17, 31]. The latter suggested that leptin may have an important therapeutic role in neurodegenerative diseases such as epilepsy. In fact, it has been established that the effects of leptin-based therapeutic strategies are potentially useful for patients with diverse diseases, besides congenital metabolic leptin deficiency disorders, such as mood disorders, Alzheimer’s disease and autism [30, 32–34].

Interestingly, some studies have explored the role of astrocytes in the activity of leptin on the CNS. These glial cells express various Ob-R subtypes. When some of them are overexpressed, changes have been demonstrated in leptin permeability through an in vitro blood brain barrier system, suggesting that astrocytes might be involved in the entrance of leptin into the CNS [35]. In mice, with specific mutations that inhibit expression of Ob-R in astrocytes (ALKO Mice-astrocyte specific leptin receptor knockout mice) studies have shown that astrocytes can intervene significantly in neuronal leptin-induced signaling [36].

Jayaram et al. [37] found that leptin may have glioprotective effects in a mouse model of seizures induced by pilocarpine administration. This model found that ALKO mice had a lower survival rate and that administration of leptin, under gliotoxicity when exposed to high levels of glutamate, exerted a protective effect on astrocytes [37]. These results suggest that leptin can have a protective role against excitotoxicity when presented during epilepsy.

At this point, it is important to note that the peripheral effects of leptin may have relevant implications on susceptibility to epilepsy. The dysfunction of the adipose tissue and the hormones that it produces, such as leptin, supposes a susceptibility to neurodegenerative diseases associated with obesity and metabolic disorders, since the neuroprotective effects that have been evidenced in the actions of leptin could be influenced by the resistance to leptin that is characteristic of disorders such as obesity, decreasing it’s neuroprotective effect and contributing to the pathogenesis of different neurodegenerative diseases, such as AD [38, 39]. In addition, obesity causes metabolic changes that alter the expression of leptin receptors in astrocytes, generating underlying alterations in the functions of glial cells that would have serious implications for leptin homeostasis as previously mentioned [36, 40]. In fact, models with adult-onset obesity mice have shown that obesity induces an increase in ObR levels in astrocytes and ObRb mRNA in the cerebral microvasculature [41], and that astrocytes play regulatory roles on the dynamics of leptin in the hypothalamus, since its inhibition improves the signaling and uptake of leptin by neurons [42]. Finally, it should be noted that obesity implies the occurrence of a sufficiently important level of peripheral inflammation, which could be mediated by the action of leptin and could contribute significantly to the development of neuroinflammation and oxidative stress in the central nervous system, increasing the susceptibility to the occurrence of neurodegenerative diseases, such as epilepsy [39, 43, 44].

It would be interesting to determine the possible effects that antiepileptic drugs (AEDs) have on the role of leptin on CNS. Weight gain is associated with changes in secretion and behavior of leptin and it is a major side effect of these drugs. It is relevant to assess whether the actions on neuronal excitability of this hormone, both positive and negative, could be involved in the evolution of epilepsy before consumption of AEDs [45–48].

## Experimental models: anticonvulsant and convulsant effects of leptin (see Additional file 1)

In recent decades, the relationship between the effect of leptin on the CNS and epilepsy has been recognized as a new and viable association that could lead to future effective alternatives to treat this disorder. Experimental animal studies conducted around this association have generated results that begin to unravel the potential therapeutic effects of leptin on metabolic and neurodegenerative diseases [1, 18, 19].

### Anticonvulsant studies

Shanley et al. [13] demonstrated in cultured hippocampal neurons of rats, previously exposed to a Mg^2+^-free medium to cause synchronous spontaneous Ca^2+^ oscillations, that application of leptin significantly reduced overall Ca^2+^ levels, in a reversible manner. To assess whether the mechanism of action of leptin depended on the activation of KATP channels or on the activation of BK channels, inhibitors of these channels were applied separately. The results showed that glybenclamide and glipizide (KATP channel inhibitors) had little effect on the ability of leptin to decrease calcium levels, while the application of iberiotoxin and charybdotoxin (BK channels inhibitors) significantly blocked the effect of leptin. Therefore, the conclusion was that leptin blocks epileptiform-like activity through activation of BK channels. This is consistent with the regulation of neuronal excitability that BK channels exert and their protective function in some types of epilepsy [49].

*In*-*vivo* studies in rodents have opened new perspectives on the therapeutic potential of leptin in epilepsy. Xu et al. [50] tested the efficacy of leptin in two seizure models. First, they demonstrated that leptin injected directly into the motor area of cerebral cortex of anesthetized 4–6 week old male rats, decreased the frequency and duration of focal seizures induced by the administration of 4-aminopyridine. In the second model, generalized seizures were induced by intraperitoneal injection of pentylenetetrazol (PTZ) in 6–8 weeks old male rats and intranasal leptin was administered. The results showed increased levels of brain and serum leptin and increased latency of convulsive seizures. Additionally, authors found that leptin inhibited synaptic response in hippocampal brain slices, in a U-shaped concentration manner and by selective inhibition of AMPA receptors.

The above results are particularly relevant because AMPA receptors mediate fast synaptic excitation and hyper-synchronization in the pathophysiology of epilepsy [51], and, as showed by Xu et al. [50], leptin inhibits synaptic AMPAergic responses by activating the JAK/PI3K pathway, suggesting that leptin could act as a good anticonvulsant agent in the treatment of epilepsy by reducing epileptiform activity, similar to the action of AMPA receptor antagonists [51].

Complementing the above results, models of epilepsy induced by PTZ demonstrated that ob/ob mice deficient in leptin have increased severity of seizures compared with wild-type mice [52]. Video electroencephalogram recordings showed an increased susceptibility to tonic–clonic and clonic generalized seizures, more deaths and a higher percentage of epileptiform activity.

In order to identify the underlying mechanisms of the anticonvulsant effects of leptin, experimental studies focused on the potential role of phosphoinositide 3 kinase (PI3K), a common intermediate of leptin signaling, downstream from the activated leptin receptors (Ob-Rb). Administration of LY294002 and wortmannin (PI3K inhibitors) reduced the effect of leptin over the decrease in spontaneous calcium oscillations and over hippocampal synaptic response, supporting the involvement of JAKs [13]. The results reinforce the idea that these intracellular pathways are important in the anticonvulsant action of leptin. Furthermore, electrophysiological studies carried out on hippocampal slices of rodents with deficiency in these receptors (Obese Zucker fa/fa rats and db/db mice) showed that leptin action was dependent on Ob-Rb when compared with slices of control rodents [13, 50].

Previous experiments have proposed neuroprotective effects of leptin in a model of status epilepticus induced by kainic acid in rodents [53]. The researchers found that leptin attenuates the degeneration of hippocampal neurons, in both acute and chronic stages. However, the effects of leptin doses were restricted only to histological results, because the occurrence of recurrent spontaneous seizures or behavioral long-term deficits could not be avoided. Interestingly, a study demonstrated that leptin treatment soon after the induction of neonatal status epilepticus in rats counteracted the long-term alterations associated to hyperexcitability, supporting leptin’s neuroprotective effects and its potential therapeutic roles [54].

Consistent with the relationship with the innate immune system, inflammation and oxidative stress in the process of epileptogenesis, Oztas et al. [55] evaluated the effect of leptin in a convulsive seizure model. They showed that the administration of leptin, in addition to causing anticonvulsant effects, reduce levels of pro-inflammatory cytokines suggesting a crucial anti-inflammatory role of leptin in epilepsy. Furthermore, leptin significantly increased the levels of galanin, a neuropeptide that has anti-excitatory properties. Regarding oxidative stress, leptin administration produced a reduction in the serum levels of malondialdehyde and an increase of glutathione, which correlates with possible protective effects against oxidative damage during epilepsy.

### Convulsant studies

Although anticonvulsants effects of leptin have been well documented, this hormone can also have convulsant actions. Using a rat model of epileptiform activity induced by penicillin, Ayyildiz found a dose-dependent effect of leptin to increase the frequency of the penicillin-induced epileptiform activity [56]. An intracerebroventricular of 1 µg of penicillin administered was the most effective dose in changing the frequency on penicillin-induced epileptiform activity.

Similarly, a study assessing the convulsant or anticonvulsant effects of leptin before the administration of glutamate or NMDA, AMPA and kainate receptor agonists in mice, showed an increased percentage of seizures, and a decreased latency in the onset of seizures in the groups that received NMDA or kainate receptor agonists together with leptin. The authors concluded that leptin exerted a convulsant activity [57].

Nitric oxide (NO) might have an essential role in epilepsy due to its properties as a second messenger, neuromodulator and neurotransmitter in the central nervous system. The literature shows an ambiguous dual action (convulsive or anticonvulsive) of NO. A kainate-induced epileptic model showed that NO end-product levels and seizure activity increased after kainate injection, and both were attenuated with the administration of the NOS inhibitor 7-nitroindazole (7-NI), suggesting that epileptic activity may be related with NO [58]. In contrast, studies that investigated the role of NO as a neuromodulator in the anticonvulsant effects of pyridoxine on penicillin-induced epileptiform activity didn’t find evidence of involvement of NOS activity in the brain, even though it was demonstrated that 7-NI by itself reduced the epileptiform activity [59]. With the aim to determine whether the convulsant effect of leptin could be related to nitric oxide, Aslan et al. induced epileptiform activity in rats through the injection of penicillin with the subsequent administration of leptin. The intervention increased significantly the frequency of epileptiform activity in relation with those animal with penicillin only [60]. In the presence of leptin, the anticonvulsant effect of 7-nitroindazole (selective inhibitor of neuronal nitric oxide synthase (NOS)) or l-arginine (nitric oxide precursor) was delayed significantly. However in the presence of NG nitro-l-arginine methyl ester (L-NAME) which is a nonselective inhibitor of NOS did not influence proconvulsant activity of leptin. This suggests that neuronal NOS pathway could effectively make part of the underlying convulsant mechanism elicited by leptin in this model. The mechanism of action of l-arginine on the effect of leptin is not fully understood. However, it has been shown previously that the administration of l-arginine alone decreases the epileptiform activity caused by penicillin, and it has been proposed that perhaps it could compete with leptin for the binding sites associated with the regulation of NO production [61, 62].

The interaction of the cannabinoid system and the effect of leptin in the CNS has been studied in relation with its anorexigenic properties and metabolic functions [63–65]. However, the anticonvulsant properties of the cannabinoid system has also been widely documented. Blair et al. showed in primary hippocampal neuronal culture models of acquired epilepsy and status epilepticus that the cannabinoid receptor agonist WIN 55,212-2 produced dose-dependent anticonvulsant effects on both models, in a CB1 receptor-dependent manner [66]. In concordance, it has been shown that administration of SR-141716A and AM-251, CB1 receptors antagonists, generated status epilepticus-like activity in a hippocampal neuronal culture model of acquired epilepsy, which is reversible with application of CB1 agonists [67]. Furthermore, in an amygdala-kindling model of temporal lobe epilepsy, Wendt et al. found that the CB1 receptor agonist WIN 55,212-2 delayed the progression of seizure severity on kindling acquisition [68]. They also found that administration of URB597, an inhibitor of the enzymatic degradation of the endocannabinoid anandamide, reduced the amount of newborn neurons during the kindling process, suggesting a disease-modifying effect of the endocannabinoid system. The involvement of cannabinoids on neuronal excitability allows to consider a possible relation with leptin effects.

Through an experimental model of epileptiform activity induced by penicillin, using electrocorticographic recordings, Arslan et al. found that administration of leptin restricted anticonvulsant activity of arachidonyl-2-chloroethylamide, a cannabinoid type 1 receptor (CB1) agonist, and increased the convulsant effect of AM-251, a CB1 receptor antagonist [69]. Therefore, the results indicated that inhibition of CB1 receptors probably mediate the convulsant effects of leptin.

## Conclusions

This paper systematically reviewed research articles that examined the association between Leptin and the Nervous system, specially its effects as anticonvulsant or proconvulsant.

Leptin can act as a neuroprotective agent that inhibits epileptiform like activity. The JAK/PI3K signaling pathway and BK channels have been associated with the anticonvulsant effects of leptin. However, more studies are required to elucidate all the intracellular pathways that leads to the mechanism of action of leptin. In concordance, chronic leptin deficiency increases brain excitability. These anticonvulsant effects of leptin have been shown in epileptic models induced by 4 aminopyridine, pentylenetrazole and in cultures exposed to stimulation in Mg^2+^ -free medium. Paradoxically, leptin has a convulsant effect, increasing the epileptiform activity after its administration, in epileptic models induced by penicillin and glutamate receptor agonists.

Anticonvulsants and convulsant effects of leptin evidenced in various experimental studies, demonstrate the need to continue studying the role of leptin in epilepsy to analyze whether certain experimental conditions could influence the results, or if instead there are intrinsic factors of the CNS that determine the effect of leptin. In this regard, it should be taken into account the conditions in which surgery was performed, the route and amount of leptin injection, and especially the length of the postoperative period before the experiments are conducted due to the fact that animals lose weight after surgery with a consequent reduction of serum leptin concentration.

The interaction between peripheral metabolic hormones, such as leptin, and higher brain functions that could influence epilepsy allows to consider potential novel therapeutic alternatives to face this neurodegenerative condition. Therefore, it is important to conduct more studies to determine the influence of leptin on the pathophysiology of epilepsy to better understand its mechanism of action and to shed light on its therapeutic potential and its possible benefits when compared with AEDs.

## Additional file

**Additional file 1.** Experimental model studies of leptin. Description of data: Comparative data of anticonvulsant and convulsant effects of leptin experimental model studies 1.

## Abbreviations

LMM: Laura Mora-Muñoz
AGN: Alejandro Guerrero Naranjo
AVM: Alberto Velez-van-Meerbeke
EARJ: Elisa Angélica Rodríguez-Jimenez
CM: Claudio Mastronardi
CNS: central nervous system
MESH: Medical Subject Headings
DeCS: Health Sciences Descriptors (Descriptores Ciencias de la Salud)
WAT: white adipose tissue
BAT: brown adipose tissue
JAK- 2: Janus kinase -2
SH2: Src homology 2 domain
ERK: extracellular signal-regulated kinase
SPH2: SH2-containing tyrosine-specific protein phosphatase
GRB2: growth factor receptor-binding protein
STAT -5: signal transducer and activator of transcription 5
STAT -3: signal transducer and activator of transcription 3
α -MSH: α-melanocyte stimulating hormone
POMC: proopiomelanocortin
SOCS3: suppressor of cytokine signaling 3
KATP channels: ATP-sensitive potassium channels
BK channels: Ca^+^-activated potassium channels
mTOR: mammalian target of rapamycin
ALKO Mice: astrocyte specific leptin receptor knockout mice
PI3K: phosphoinositide 3 kinase
NO: nitric oxide
7-NI: NOS inhibitor 7-nitroindazole
NOS: neuronal nitric oxide synthase
