# Supplementary material for: Leptin: role over central nervous system in epilepsy
Source: BMC Neurosci. 2018 Sep 5;19:51. doi: 10.1186/s12868-018-0453-9 (PMC6126011; doi:10.1186/s12868-018-0453-9)
Supplement: Supplementary file 1 — Additional file 1. Experimental model studies of leptin. Description of data: Comparative data of anticonvulsant and convulsant effects of leptin experimental model studies 1. [file 12868_2018_453_MOESM1_ESM.docx]

| ARTICLE | EPILEPTOGENIC AGENT | SUBJECTS | EXPERIMENTAL CONSIDERATIONS | ADMINISTRATION ROUTES* | GENERAL REMARKS | LEPTIN EFFECT |
| --- | --- | --- | --- | --- | --- | --- |
| Shanley LJ, O’Malley D, et al. 2002 | Mg^2+^-free médium | 1-3 days old rat pups (For culture of hippocampal neurons).  3-4-week-old -Zucker rats (Lean rats and obese fa/fa rats) (For rat hippocampal slices). | *In-vitro study.*  Cultures of hippocampal neurons were used to evaluate the effect of leptin over intracellular Ca^2+^ levels (Using a digital epifluorescence imaging system) and to determine de functional localisation of leptin receptors with immunocytochemical techniques.  Rat hippocampal slices were used to determine if leptin modulates the epileptiform-like activity, using electrophysiological recordings. | Does not apply | Leptin inhibits epileptiform-like activity via PI3-kinase-driven activation of BK channels.  Leptin modulates epileptiform-like activity via leptin receptor activation. | Anticonvulsant effect |
| Xu L, Rensing N, et al. 2008 | 4-aminopyridine (4-AP)  Pentylenetetrazole (PTZ) | 4-6-week-old male Sprague-Dawley rats (For neocortical [4-AP] seizure model)    6-8-week-old male CD-1 mice (For PTZ generalized seizure model)  Swiss-Webster mice (Tissue preparation for electrophysiology)    Male db/db and C57BLKS/J mice (Assessment of leptin effects in db/db mice) | *In-vivo study*  Mice were anesthetized with halothane.  EEG recordings were used to evaluate the effect of leptin in the seizure models.  A reviewer blinded to the experimental protocols analyzed the EEG recordings.  A commercial mouse ELISA kit was used to measure serum and brain leptin levels.  *In-vitro study*  Cells of hippocampal cultures and hippocampal slices were used for electrophysiological analysis, using patch-clamp recordings.  Mice were killed under halothane. | 4-AP was administered by a direct cortical injection.  Leptin was administered by a direct cortical injection (In the neocortical [4-AP] seizure model) and intranasally (In the PTZ generalized seizure model)  PTZ was administered intraperitoneally | Leptin shortens and reduces 4-AP-induced seizures in rats.  Intranasal leptin administration delays the seizure latency in PTZ-induced seizures.  Intranasal leptin administration increases serum and brain leptin levels.  Leptin inhibits AMPAR-synaptic transmission in mice via JAK2/PI3K signaling pathway, and requires Ob-Rb activation. | Anticonvulsant effect |
| Erbayat-Altay E, Yamada KA, et al. 2008 | Pentylenetetrazole (PTZ) | 44-58-day-old leptin deficient ob/ob male mice  44-58-day-old wild type (C57BL/6J) male mice | *In-vivo study*  Mice were anesthetized with halothane.  Continuous EEG recordings were used to evaluate the seizure activity in ob/ob and wild type male mice. | PTZ was administered intraperitoneally | There is an Increased severity of PTZ-induced seizures in leptin deficient ob/ob mice compared with wild type mice. | Anticonvulsant effect |
| Obeid M, Frank J, et al. 2010 | Kainic acid (KA) | Adult male Sprague–Dawley rats | *In-vivo study*  Intermittent video monitoring was used to evaluate recurrent spontaneous seizures.  Two reviewers blind to treatments analyzed the video recordings.  Behavioral tests were performed to evaluate the acute and long-term behavioral deficits.  *In-vitro study*  Histological analysis was used to evaluate the neuroprotection effect of leptin in acute and long-term. | KA was administered intraperitoneally  Leptin was administered by intraperitoneal injections | Leptin has a neuroprotective effect against KA-induced hippocampal damage.  Leptin does not prevent long-term recurrent spontaneous seizures  Leptin does not prevent acute and long-term behavioral deficits. | Neuroprotective effect |
| Oztas B, Sahin D, et al. 2017 | Pentylenetetrazole (PTZ) | Wistar – Albino Rats | *In-vivo study*  EEG records and behavioral changes were monitored to evaluate the epileptic activity.  Biochemical studies were carried out to evaluate the serum levels of pro-inflamatory cytokines, galanin, nitric oxide, malondialdehyde and glutathione. | PTZ was administered intraperitoneally  Leptin was administered intraperitoneally | Leptin delays seizure onset and reduces seizure severity.  Leptin reduces pro-inflamatory cytokines levels, increases galanin levels and improve the oxidant/antioxidant parameters | Anticonvulsant effect |
| Ni H, Chen SH, et al. 2017 | Pilocarpine | Sprague-Dawley Rats on postnatal day 5 | *In-vivo study*  Morris water maze test was used to evaluate learning and memory abilities.  Seizure susceptibility was evaluated through seizure latency records.  Timm staining was used to evaluate mossy fiber sprouting.  Western Blot analysis was carried out to evaluate the ZnT3 and CB-D28k protein levels in the hippocampus. | Pilocarpine was administered intraperitoneally  Leptin was administered intraperitoneally | Seizure thresholds were higher in the group of pilocarpine-induced status epilepticus plus leptin compared with the group without administration of leptin.  Leptin treatment soon after induction of status epilepticus counteracted long-term alterations. | Neuroprotective effect |
| Ayyildiz M, Yildirim M, et al. 2005 | Penicillin | Female Wistar rats | *In-vivo study*  Rats were anesthetized with urethane.  Rats blood pressure was kept above 100 mmHg during experiments.  The electrocorticographic activity was monitored to analyze the epileptic activity | Penicillin was administered by an intracortical injection  Leptin was administered by an intracerebroventricular injection | Leptin increases the frequency of penicillin-induced epileptic activity | Proconvulsant effect |
| Lynch III JJ, Shek EW, et al. 2010 | Glutamate and selective agonists of glutamate receptor subtypes: NMDA, AMPA and kainate | 8-10-weeks-old male C57BL/6JRj mice | *In-vivo study*  All testing was performed during approximately the same time of the day.  Continuous observation was performed to evaluate behavioral signs. | Leptin was administered intraperitoneally  Glutamate and selective agonists of glutamate receptor subtypes were administered intraperitoneally | Leptin exhibits dose-related proconvulsant activity on NMDA- and kainite-mediated seizures in mice, as was observed on behavioral signs of convulsions.  Leptin does not have clear convulsant-related effects with glutamate and AMPA agonist. | Proconvulsant effect |
| Aslan A, Yildrim M, et al. 2010 | Penicillin | Male Wistar rats | *In-vivo study*  Rats were anesthetized with urethane.  Rats blood pressure was maintained above 110 mmHg during the experiments.  The electrocorticographic activity was continuously monitored to evaluate the epileptiform activity.  All recordings were made under anesthesia. | Penicillin was administered by intracortical injections.  Leptin was administered intracerebroventricularly.  Nitric oxide synthase inhibitors were administered intraperitoneally. | Neural NOS/NO pathway is involved in mediating leptin proconvulsant effects on penicillin-induced epileptiform activity.  The results imply that neuronal NOS, but not endothelial NOS, participates in the proconvulsant effect of leptin. | Proconvulsant effect |
| Arslan G, Alici S, et al. 2013 | Penicillin | Male Wistar rats | *In-vivo study*  Rats were anesthetized with urethane.  The electrocorticographic activity was continuously monitored and recorded to analyze the epileptiform activity. | Penicillin was administered by intracortical injections.  Leptin was administered intracerebroventricularly.  CB1 agonist and antagonist were administered intracerebroventricularly. | Proconvulsant activity of leptin is mediated, in part, by inhibition of cannabinoids in penicillin-induced epilepsy. | Proconvulsant effect |

*Evaluated only for in-vivo studies.
